# Screening UFMylation-associated genes in heart tissues of Ufm1-transgenic mice

**DOI:** 10.1186/s12872-023-03563-7

**Published:** 2023-11-18

**Authors:** Hu Jiajia, Yang Ziyao, Zheng Jiaqi, Chen Yanli, Zhao Xiaotao, Su Ming

**Affiliations:** 1 Department of Clinical Laboratory, Peking University People’s Hospital, No.11 Xizhimen South Street, Xicheng District, Beijing, 100044 China; 2 Department of Blood Transfusion, Peking University First Hospital, Beijing, China; 3 State Key Laboratory of Vascular Homeostasis and Remodeling, Peking University, Beijing, China

**Keywords:** Ufm1, UFMylation, Cardiac hypertrophy, Tnfaip2

## Abstract

UFMylation is a ubiquitination-like modification that is related to endoplasmic reticulum stress and unfolded protein response. A recent study reported that Ufl1, a key enzyme of UFMylation, protects against heart failure, indicating that UFMylation may be associated with heart function regulation. In the present study, we initially constructed a Flag-6×His-tagged Ufm1ΔSC transgenic (Tg-Ufm1) mouse model that enables UFMylation studies in vivo. Tg-Ufm1 mice showed significant activation of UFMylation in hearts. By using this model, we identified 38 potential Ufm1-binding proteins in heart tissues through LC‒MS/MS methods. We found that these proteins were associated with mitochondria, metabolism and chaperone binding. By using transcriptomic screening, we identified Tnfaip2 as a novel UFMylation-associated gene. Overexpression of Ufm1 significantly upregulated the protein expression of Tnfaip2, whereas isoproterenol treatment decreased Tnfaip2 expression in Tg-Ufm1 mice. These data may provide novel clues for UFMylation in cardiac hypertrophy.

**Supplementary Information:**

The online version contains supplementary material available at 10.1186/s12872-023-03563-7.

## Introduction

Cardiac hypertrophy is an important cardiovascular response that significantly affects human health. There are two types of cardiac hypertrophy: physiological and pathological, both of which initially develop as an adaptive response to stress. Physiological hypertrophy maintains cardiac function, whereas long-term pathological hypertrophy usually develops into heart failure and is a leading cause of morbidity and mortality worldwide [1, 2]. It has been reported that the prevalence of hypertrophic cardiomyopathy, an inherited left ventricular hypertrophy disease, in the general adult population ranges from 1:500 to as high as 1:200 [3–8]. Studies have revealed that multiple factors, including hereditary and nonhereditary factors, can induce cardiac hypertrophy [1, 2, 9]. Nevertheless, the mechanisms involved in the development of cardiac hypertrophy have not been fully elucidated. Stress, as a main risk factor for the development of cardiac hypertrophy, has been reported in many studies [9–13]. Protein synthesis in hypertrophic hearts is increased, which may more easily induce endoplasmic reticulum (ER) stress and the unfolded protein response (UPR) [14–16]. Therefore, protein quality control (PQC) is crucial in maintaining the normal function of the heart during hypertrophy. Recently, Li et al. reported that the Ufm1-specific ligase Ufl1 protects against heart failure by maintaining ER homeostasis [17], indicating that UFMylation may participate in cardiac remodeling. Therefore, the identification of UFMylation-associated genes in hearts may provide novel clues for further investigation.

Although UFMylation has been discovered for decades, most of the studies are based on in vitro models, and an ideal in vivo model is still lacking. In the present study, we constructed a Flag-6×His-tagged Ufm1ΔSC transgenic mouse model, which enables UFMylation investigation in vivo. By using this model, we analyzed UFMylation-associated genes with proteomic and transcriptomic methods.

## Materials and methods

### Animal study

The animal experimental procedures were approved by the Peking University People’s Hospital Committee for Animal Use (No. 2018PHC051). Tandem Flag-6×His-tagged Ufm1ΔSC (Ufm1 lacking the C-terminal two amino acid residues) global transgenic (Tg-Ufm1) mice were generated as previously described [18]. Eight- to ten-week-old Tg-Ufm1 mice and their nontransgenic (NTg) littermates were used in this study. To induce cardiac hypertrophy in mice, 5 mg/kg body weight/day isoproterenol (ISO) was administered through subcutaneous injection on the back for five days. On the sixth day after injection, the mice were anaesthetized by inhalation of isoflurane and then killed by cervical dislocation, and the hearts were harvested for use. The heart weight/body weight (HW/BW, mg/g) ratio was measured.

### RNA extraction and quantitative PCR analysis

Heart tissues were lysed in RNAiso Plus (Takara Bio, Dalian, China), and total RNA was extracted using a routine chloroform-isopropanol method. RNA samples were quantified and normalized. cDNA was synthesized by using PrimeScript™ RT Master Mix (Takara), and real-time qPCR was performed with Hieff® qPCR SYBR Green Master Mix (Yeasen, Shanghai, China). Primer sequences are listed as follows: Tnfaip2, 5′-GCCATCGACATTCTCCAGATCA-3′ and 5′-ATCAAAGGCGCGCTGGTAG-3′; Esyt2, 5′-CTTTCGAGAAACCATAGAGCCAG-3′ and 5′-CCTTAACACCATTGACTCTCAGG-3′; Mrpl48, 5′-CCAGCATCCAGGAGCTTGAA-3′ and 5′-AGCCCATGTTGTATCCAGCC-3′; Gpsm1, 5′-TGCGGCACCTAGTCATTGC-3′ and 5′-TGTCAGTTCTCCGTTTCGGTC-3′; Pcbp4, 5′-CCCGAGCTTAGTATCACCCTC-3′ and 5′-CCCGGATTCGCTTTACAGTCT-3′; Myadm, 5′-ATGCCGGTAACAGTAACTCGT-3′ and 5′-CCACACAGGTGGATATTAGCTG-3′;β-actin, 5′-CTCAGGAGGAGCAATGATCTTGAT-3′ and 5′-TACCACCATGTACCCAGGCA-3′; Anp, 5′-TCTTCCTCGTCTTGGCCTTT-3′ and 5′-CCAGGTGGTCTAGCAGGTTC − 3′; Bnp, 5′-TGGGAGGTCACTCCTATCCT − 3′ and 5′-GGCCATTTCCTCCGACTTT − 3′; Acta1, 5′-CCCAAAGCTAACCGGGAGAAG − 3′ and 5′-GACAGCACCGCCTGGATAG − 3′;TNF-α, 5′-GTCCCCAAAGGGATGAGAAGT-3′ and 5′-TTTGCTACGACGTGGGCTAC − 3′;Ufm1, 5′-GCTGCCGTACAAAGTTCTCA-3′ and 5′-CCAGCAGTCTGTGCAGGATT-3′;RPL26, 5′-AGAACCGCAAACGGCATT TC-3′ and 5′-GTCCGCGAACAACCTGAA CT-3′. The relative expression was calculated using the 2-^ΔΔCt^ method, and β-actin was used as an internal control for normalization.

### Transcriptomic analysis

A total of 1 µg of RNA from each sample was used for transcriptomic sequencing. The libraries were generated using a VAHTSTM mRNA-seq V2 Library Prep Kit for Illumina® according to the manufacturer’s instructions. The library quality was assessed on the Agilent Bioanalyzer 2100 system, quantified and pooled. Paired-end sequencing of the library was performed on NovaSeq sequencers (Illumina, San Diego, CA). FastQC (version 0.11.2) was used to evaluate the quality of the sequenced data. Raw reads were filtered by Trimmomatic (version 0.36). Clean reads were mapped to the reference genome by HISAT2 (version 2.0), and RSeQC (version 2.6.1) was used to calculate the alignment results. Gene expression values of the transcripts were determined using StringTie (version 1.3.3b). DESeq2 (version 1.12.4) was used to determine DEGs. DEGs were defined as those with a q value ≤ 0.05 and |FoldChange| ≥ 2. Functional enrichment analyses, including Gene Ontology (GO) and KEGG, were performed.

### Protein extraction and western blotting

Heart tissues were lysed with RIPA lysis buffer containing protease and phosphatase inhibitors. The samples were quantified and diluted to 1 µg/µL and denatured, and equal amounts of protein were loaded onto an SDS‒PAGE gel for separation. The proteins were then blotted onto nitrocellulose membranes. The membranes were cropped according to the molecular weight of target proteins (except for Ufm1) with the reference of the manufacturers’ instructions, whereas full length membranes were used for the detection of Ufm1. The membranes were blocked with 5% nonfat milk at room temperature for 1 h and incubated with the following primary antibodies at 4 °C overnight: rabbit anti-Ufm1 (Abcam), and rabbit anti-β-Tubulin (Proteintech, Wuhan, China), rabbit anti-Uba5 (Proteintech, Wuhan, China), rabbit anti-Ufc1 (Proteintech, Wuhan, China), rabbit anti-Ufl1 (Proteintech, Wuhan, China), mouse anti-Tnfaip2 (Santa Cruz Biotechnology Inc., Dallas, TX, USA). The membranes were then washed four times with TBST and incubated with an HRP-conjugated goat anti-rabbit or anti-mouse secondary antibody (ZSGB-BIO, Beijing, China) for 1 h at room temperature. Then, the membranes were washed with TBST five times and stained with Super Signal West Femto Maximum Sensitivity Substrate (Pierce, Rockford, IL, USA). The bands were visualized, and the intensity of each band was scanned with Quantity One software V 4.6.2 (Bio-Rad, Hercules, CA, USA). The relative expression was calculated by normalizing to β-Tubulin.

### Pulldown assay

Heart tissues were lysed with NP-40 lysis buffer containing protease and phosphatase inhibitors. Briefly, the samples were homogenized on ice and centrifuged 8000 times per minute (rpm) at 4 °C. The supernatants were quantified and diluted to 1 µg/µL, and an equal volume of lysates was incubated with Dynabeads™ His-Tag Isolation and Pulldown (Thermo Fisher, Carlsbad, CA, USA). The lysate-bead mixture was continuously suspended with a vertical mixer at 4 °C for 30 min. Then, the beads were separated and washed five times and eluted with 300 mM imidazole solution.

### Proteomic analysis

The elution products from the pulldown assay were purified and separated with an EASY-nLC1200 coupled to a Q-Exactive mass spectrometer (MS) (Thermo Finnigan). The separation procedure was performed using a reversed-phase column (100 μm, ID × 15 cm, Reprosil-Pur 120 C 18 -AQ, 1.9 μm, Dr. Math). The mobile phases were H2O with 0.1% FA, 2% acetonitrile (phase A), 80% acetonitrile, and 0.1% formic acid (phase B). A 120 min gradient at a 300 nL/min flow rate was used for sample separation. Gradient B was 8 to 35% for 92 min, 35 to 45% for 20 min, 45 to 100% for 2 min, 100% for 2 min, 100 to 2% for 2 min and 2% for 2 min. Data-dependent acquisition was performed with an Orbitrap analyzer at a resolution of 70,000 (m/z 200) for MS1, and the resolution was 35,000 for MS2. The automatic gain control target for MS1 was 1.0E + 6 and for MS2 was 1.0 E + 5. The 10 most intense ions were fragmented by HCD with a normalized collision energy (NCE) of 28%, and the isolation window was 2 m/z. The dynamic exclusion time window was 30 s. Raw MS files were processed and quantified with MaxQuant (Version 1.6.1.0). The UniProt-mouse-20,200,526.fasta database was searched using MaxQuant software. Trypsin/P was set as a specific enzyme with up to 2 missed cleavages. Carbamidomethyl on cysteine was identified as a fixed modification. Oxidation of methionine and acetyl on the protein N-terminus were identified as variable modifications. The FDR should be less than 0.01. Only the unique and razor peptides were used for quantification, and the other parameters were reserved as default. The peptides in the control were filtered from the test group, and the peptides or proteins with a unique peptide number ≥ 2 in the test group were included. Enrichment analyses, including Gene Ontology (GO) and KEGG, were performed. The STRING database was used for protein‒protein interaction analysis.

### Statistical analysis

Data are presented as the mean ± standard deviation (SD). The statistical procedures were performed using GraphPad Prism 9.0 (GraphPad Prism Software, Inc.). Differences between groups were tested using Student’s t test or two-way analysis of variance. P < 0.05 was defined as statistically significant.

## Results

### Screening for Ufm1 binding proteins in heart tissues

**Fig. 1:**
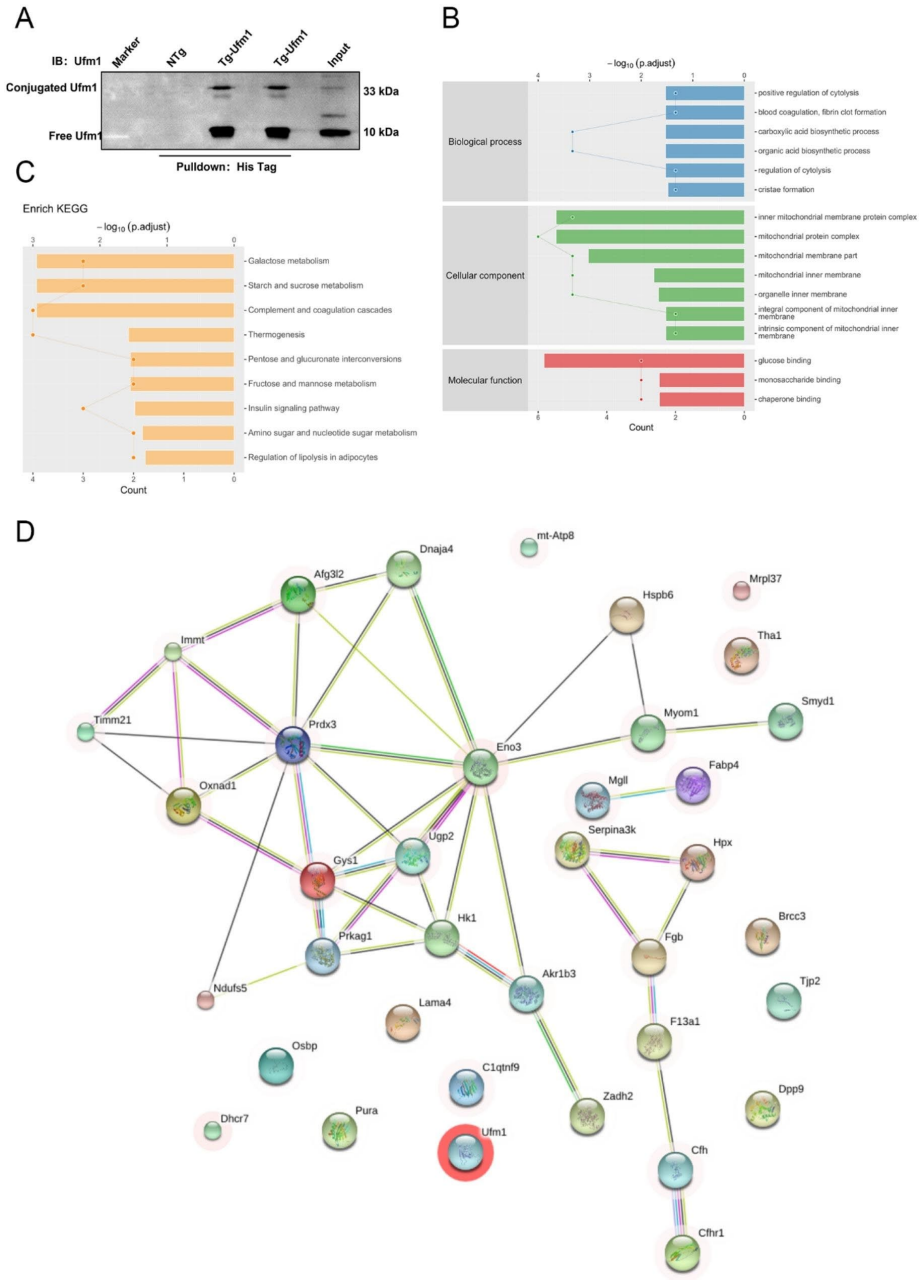
Screening of Ufm1-binding proteins in heart tissues. **(A-D)** A pulldown assay was performed with Tg-Ufm1 and NTg (as a control) mice by using His-Tag affinity beads. The bands were immunoblotted with an antibody against Ufm1 **(A)**. GO **(B)**, KEGG **(C)** and protein‒protein interaction **(D)** analyses were performed

UFMylation is a process that is mediated by enzymes (Uba5, Ufc1 and Ufl1) to promote the conjugation of Ufm1 to the target proteins. To explore the action of UFMylation in heart tissues, we initially screened Ufm1-binding proteins in vivo. Global Tg-Ufm1 mice expressing 6×His-tagged Ufm1ΔSC were constructed, and the potential Ufm1-binding proteins were pulled down for proteomic screening (Fig. 1A). By filtering the proteins in the control, we identified 38 proteins as potential Ufm1 binding candidates (Supplemental Table 1). GO and KEGG enrichment analyses indicated that these proteins were enriched for mitochondrial membrane components, metabolism and chaperone binding (Fig. 1B C): Ndufs5, Immt, mtATP8, Timm21, Afg312 and Mrpl37 were enriched in mitochondrial components; Hspb6, Fgb and Dnaja4 were enriched in chaperone binding factors; Cfh and Cfhr1 were enriched in regulation of cytolysis; and Gys1, Ugp2, Mgll, Hk1, Prkag1, Eno3 and Tha1 were enriched in metabolism. The interaction network is shown in Fig. 1D.

### Ufm1 overexpression activates UFMylation in hearts

**Fig. 2:**
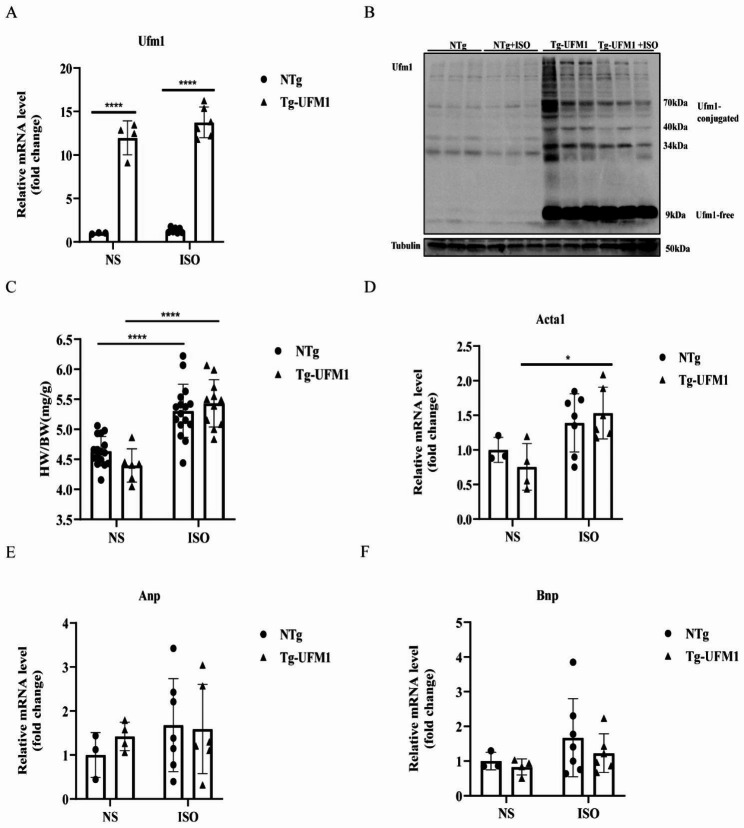
Ufm1 overexpression activates UFMylation in cardiac tissues of mice. **(A, B)** Tg and NTg mice were treated with isoproterenol (ISO) or normal saline (NS). qRT-PCR was performed to analyse the transcript levels of Ufm1 **(A).** The protein expression of Ufm1 was detected using western blotting **(B)**, and the relative expression was calculated by normalizing to β-Tubulin. *P < 0.05 compared between the indicated groups. **(C)** The heart weight/body weight (HW/BW) ratio in mice as indicated. **(D-F)** The mRNA levels of Acta1 **(D)**, Anp **(E)** and BNP **(F)** were detected using qRT-PCR. Data are shown as mean ± standard deviation *P < 0.05, **P < 0.01, and ****P < 0.0001 between the indicated groups

ISO is a known pro-hypertrophic factor by activation of adrenergic receptors. To induce cardiac hypertrophy, ISO was administered to Tg and NTg mice. Tg-Ufm1 mice showed 11.98 and 9.52 fold increase of Ufm1 expression at transcript levels in the absence and presence of ISO treatment (Fig. 2A), and 51.91 and 13.33 fold increase at protein levels (Fig. 2B). In addition, ISO treatment failed to vary Ufm1 expression in NTg mice (Fig. 2A and B).

To analyse the effects of Ufm1 overexpression on cardiac hypertrophy, we calculated the HW/BW ratio, hypertrophy and heart failure biomarkers. We found that 5 mg/kg body weight of ISO treatment for 5 days was sufficient to increase the HW/BW ratio and expression of hypertrophy biomarker Acta1, but did not increase heart failure biomarkers, such as Anp and Bnp (Fig. 2C–F). However, overexpression of Ufm1 in hearts failed to vary ISO-induced HW/BW ratio (Fig. 2C) and Acta1 increase (Fig. 2D), indicating that Ufm1 overexpression were not able to vary ISO-induced cardiac hypertrophy.

### The expression of UFMylation enzymes

**Fig. 3:**
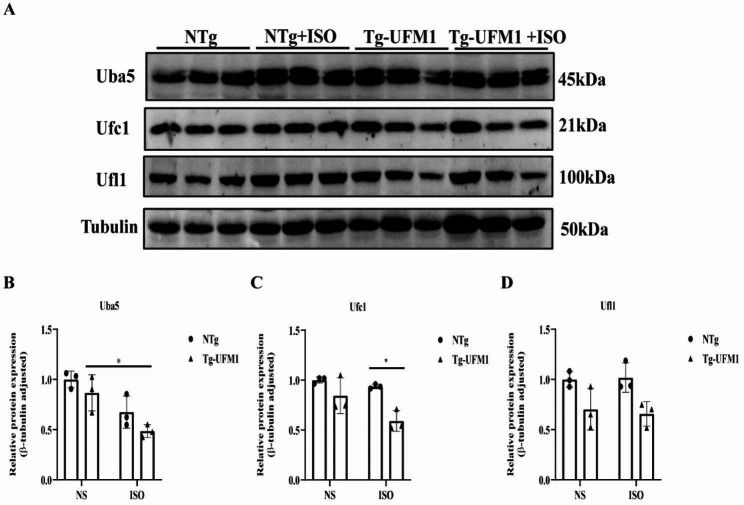
The expression of UFMylation enzymes in heart tissues. **(A-D)** The expression of Uba5, Ufc1 and Ufl1 was detected using western blotting **(A)**, and the relative expression was calculated by normalizing to β-Tubulin **(B-D)**. *P < 0.05 compared between the indicated groups

UFMylation is mediated by three main enzymes: Uba5 (E1), Ufc1(E2) and Ufl1(E3). Although ISO was not able to activate UFMylation in heart tissues, the changes of UFMylation enzymes under ISO treatment remains unclear. Therefore, we detected these enzymes in heart tissues of NTg and Tg-Ufm1 mice, respectively. We found that ISO treatment was not able to vary Uba5, Ufc1 and Ufl1 in NTg mice (Fig. 3A–D). In contrast, ISO induced a significantly decrease of Uba5 expression in Tg-Ufm1 mice (Fig. 3A and B). We observed that the expression of Ufc1 was downregulated in Ufm1-Tg compared with NTg mice in the presence of ISO (Fig. 3A and C). Together with our data in Fig. 2B, we postulate that ISO is not able to activate cardiac UFMylation sufficiently, although some enzymes’ expression of UFMylation can be slightly changed by ISO.

### Transcriptomic screening for UFMylation-associated genes

We then screened UFMylation-associated genes in Tg-UFM1 mice with or without ISO treatment by using transcriptomic sequencing. The overall transcriptomic signature is shown in Supplemental Fig. 1A. By comparing DEGs between Tg and NTg mice, we found no DEGs at baseline, and 10 DEGs were found in ISO-induced hypertrophic hearts, including 5 upregulated and 5 downregulated DEGs (Supplemental Fig. 1B; Supplemental Table 2). In contrast, ISO treatment induced 1698 DEGs in NTg mice and 1487 DEGs in Tg mice, including 1181 overlapping DEGs (Supplemental Fig. 1C-1E; Supplemental Tables 3 and 4).

**Fig. 4:**
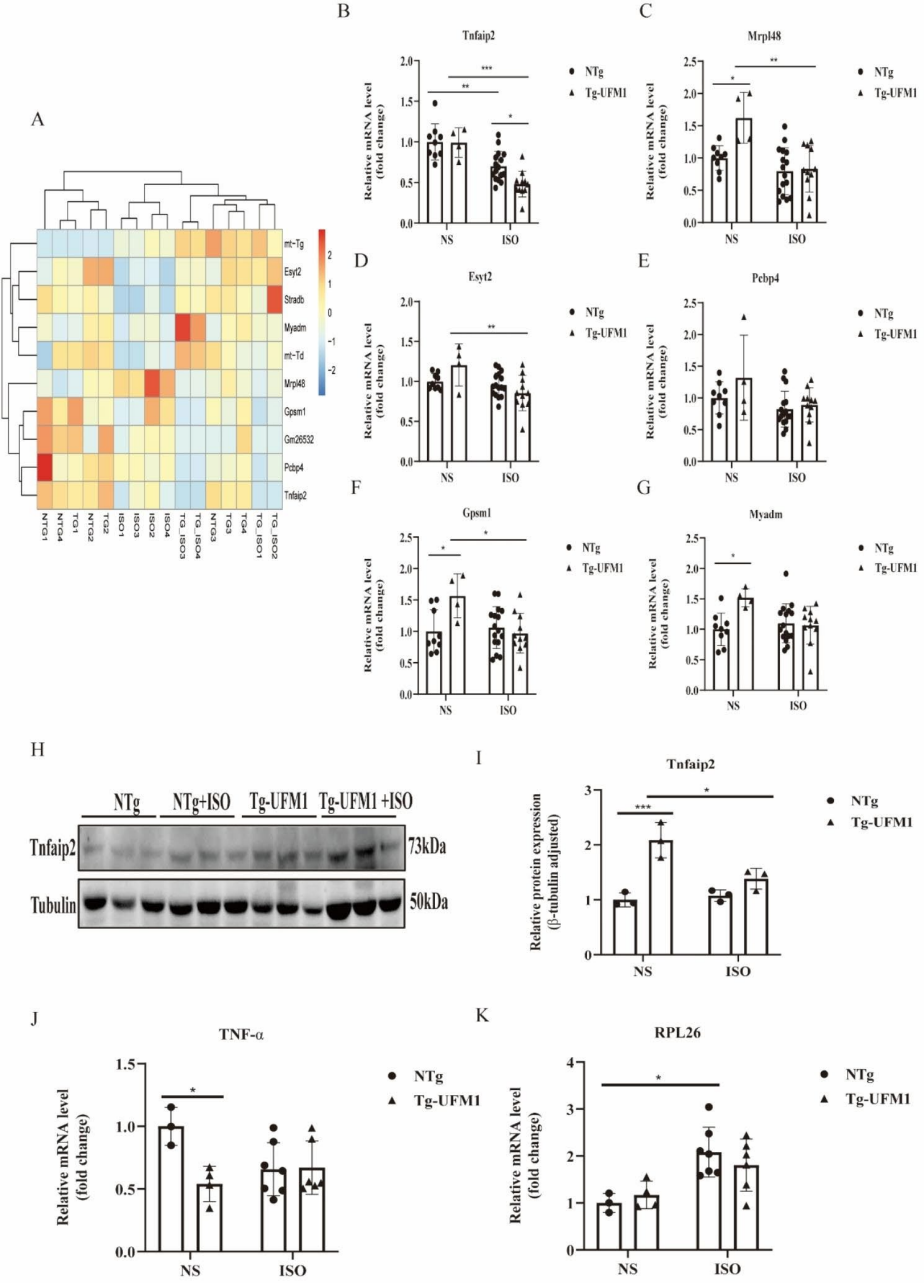
Screening and validation of UFMylation-associated genes in heart tissues. **(A-G)** The differentially expressed genes (DEGs) in heart tissues were analysed. The candidate DEGs from transcriptomic screen are shown as a Heat map (A), and the relative expression of the indicated genes in mice was validated using a real-time qPCR assay **(B-G)**. The data are expressed as the mean ± standard deviation. Two-way ANOVA was used to test significance. *P < 0.05, **P < 0.01, ***P < 0.001 and ****P < 0.0001 between the indicated groups, n = 9 in NTg mice treated with NS, 4 in Tg mice treated with NS, 16 in NTg mice treated with ISO and 11 in Tg mice treated with ISO. **(H, I)** The expression of Tnfaip2 was detected using western blotting **(H)**, and the relative expression was calculated by normalizing to β-Tubulin **(I)**. **(J, K)** The expression of TNF-α **(J)** and Rpl26 **(K)** were detected using qRT-PCR. *P < 0.05, **P < 0.01 compared between the indicated groups

To explore the effects of Ufm1 overexpression on different expressed genes (DEGs) in hypertrophic hearts, we selected 6 in 10 total DEGs from NTg vs. Tg mice in the presence of ISO (Fig. 4A), including Tnfaip2, Esyt2, Mrpl48, Gpsm1, Pcbp4 and Myadm. Real-time qPCR was performed in an expanded sample size. We found that Tnfaip2 was consistent with the transcriptomic data, whereas the other 5 genes were not (Fig. 4B–G). We observed that Tnfaip2 can be downregulated by ISO treatment in both Tg and NTg mice, and its expression in Tg-Ufm1 mice treated with ISO was lower than that in NTg mice (Fig. 4B), indicating that Tnfaip2 is a UFMylation-associated gene in ISO-induced cardiac hypertrophy.

### Expression of Tnfaip2 and Rpl26 in heart tissues of mice treated with ISO

Tnfaip2 is a TNFα-induced gene that has not been previously reported to be associated with cardiac disorders. Therefore, we next detected its protein expression in the heart tissues of mice treated with ISO (Fig. 4H). Our data showed that overexpression of Ufm1 in mice significantly upregulated Tnfaip2 protein in the absence of ISO treatment (Fig. 4I). However, Tnfaip2 protein can be downregulated by ISO treatment in Tg-Ufm1 mice, which is consistent with its expression at transcript level (Fig. 4B). As Tnfaip2 is a TNFα-induced gene, we detected the expression of TNFα. We found that overexpression of Ufm1 slightly decreased the transcript levels of TNFα, whereas its expression were comparable in the presence of ISO (Fig. 4J). In addition, the expression of Rpl26, a known substrate of UFMylation, was upregulated by ISO in NTg mice rather than in Tg-Ufm1 mice(Fig. 4K).These data indicated that Tnfaip2 may be a novel UFMylation-related gene that is associated with cardiac hypertrophy.

## Discussion

UFMylation is reported to participate in ER stress and PQC, which is associated with many diseases [17, 19–24]. The UFMylation-associated genes are not clear, especially in heart tissues. In the present study, we screened Ufm1-binding proteins in Tg-Ufm1 mice and analyzed the DEGs in the heart tissues of a tagged Ufm1 Tg mouse model. We found 38 potential Ufm1-binding proteins and identified Tnfaip2 as a candidate UFMylation-associated gene in cardiac hypertrophy, which may provide novel evidence for further research on UFMylation in cardiac hypertrophy in vivo.

Tnfaip2 has been reported to be associated with cancers [25, 26] and inflammation [27, 28], which can be induced by tumor necrosis factor alpha (TNFα) [29]. However, Tnfaip2 has not been reported to be related to cardiac hypertrophy until recently. We found that Ufm1 overexpression significantly upregulated the protein expression of Tnfaip2 and ISO treatment inhibited its upregulation. However, the expression of Tnfaip2 protein was not paralleled with its transcript, indicating that UFMylation might participate in Tnfaip2 regulation at the post-transcriptional level. Recently, Tnfaip2 was shown to participate in stem cell differentiation and organ homeostasis by controlling lipid metabolism [30]. Whether ISO-induced downregulation of Tnfaip2 is associated with cardiac disorder remains to be investigated.

Although UFMylation has been discovered for decades, the substrates and their biological functions remain far from understood. UFMylation is a ubiquitination-like modification that is mediated by enzymes catalyzing the conjugation of Ufm1 to substrates [19]. In the present study, we performed experiments using an ISO-induced compensatory cardiac hypertrophy model, representing an early stage of cardiac hypertrophy. Our data indicated that ISO treatment is not able to activate UFMylation in heart tissues. The activation of UFMylation mostly relies on the changes of the UFMylation enzymes, including Uba5, Ufc1 and Ufl1 [31]. Our results indicated that ISO is not able to increase these enzymes in heart tissues, supporting the conclusion that ISO-induced compensatory cardiac hypertrophy may not be associated with UFMylation. However, whether UFMylation can be activated in decompensatory cardiac hypertrophy or heart failure stage remains unclear.

Recent studies of UFMylation mostly rely on in vitro models. To our knowledge, this is the first study analyzing Ufm1-binding proteins in vivo. Our Flag-6×His-tagged Ufm1ΔSC transgenic mice showed activation of UFMylation in heart tissues and can be used to analyze UFMylation and Ufm1-interaction proteins in vivo. In heart tissues, we found 38 candidate proteins. GO enrichment analysis indicated that Ndufs5, Immt, mtATP8, Timm21, Afg312 and Mrpl37 are mitochondrial components, indicating that UFMylation may be associated with mitochondrial function in heart tissues. A recent report demonstrated that P4HB UFMylation regulates mitochondrial function [32] and enhances the relationship between UFMylation and mitochondria. However, the proteins identified in the present study are Ufm1-binding proteins rather than UFMylation substrates, and we did not perform validation experiments in the present study. Therefore, future studies should focus on the validation of UFMylation substrates in vivo.

## Conclusions

In conclusion, the present study constructed an available animal model that can be used for UFMylation experiments in vivo, and our data may provide novel clues for the relationship between UFMylation and cardiac hypertrophy for future study.

### Electronic supplementary material

Below is the link to the electronic supplementary material.

Supplementary Material 1

Supplementary Material 2

Supplementary Material 3

Supplementary Material 4

Supplementary Material 5

Supplementary Material 6

Supplementary Material 7

Supplementary Material 8

## Data Availability

The datasets generated and analysed during the current study are available in the Sequence Read Archive (SRA; https://dataview.ncbi.nlm.nih.gov/object/PRJNA967505?reviewer=jq21hhu1tbjqkthvggcgjb8sn4) database (PRJNA967505) and iProX database (https://www.iprox.cn/page/PSV023.html;?url=1684392080938O9UD).
