# Supplementary material for: Screening UFMylation-associated genes in heart tissues of Ufm1-transgenic mice
Source: BMC Cardiovasc Disord. 2023 Nov 18;23:567. doi: 10.1186/s12872-023-03563-7 (PMC10657630; doi:10.1186/s12872-023-03563-7)

Figure 1A: Rabbit monoclonal to ufm1 （Free ufm1 10 kDa）

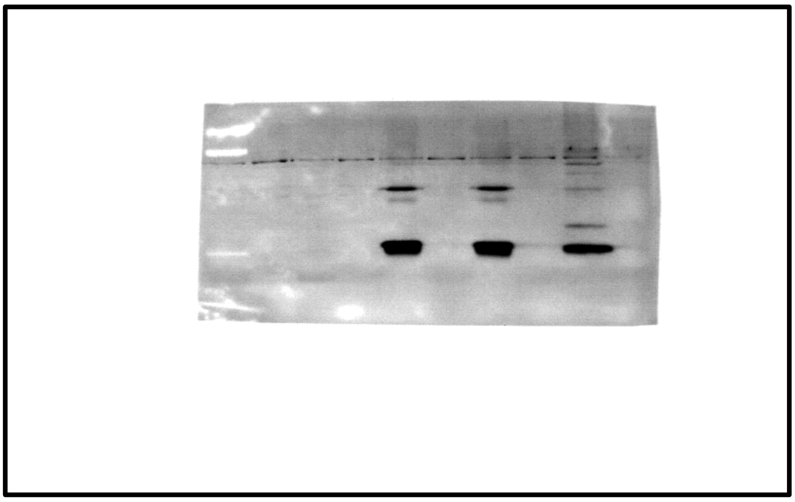

Figure 2A: Rabbit monoclonal to ufm1 （Free ufm1 10 kDa）

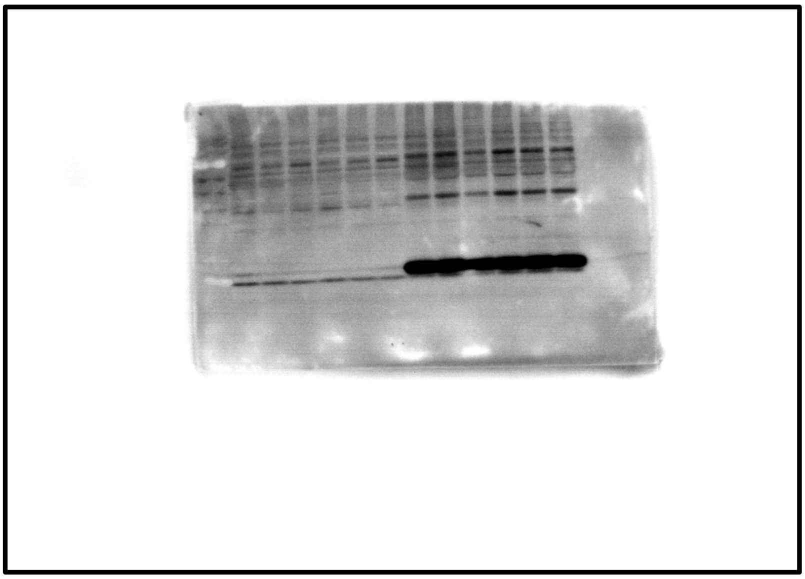

Figure 2A: Rabbit monoclonal to GAPDH（37 kDa）

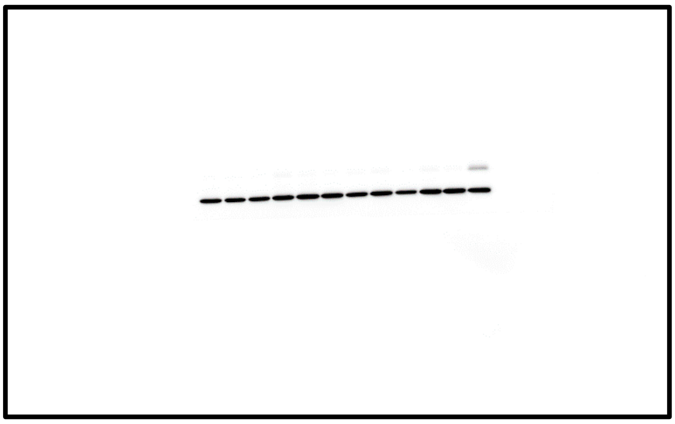 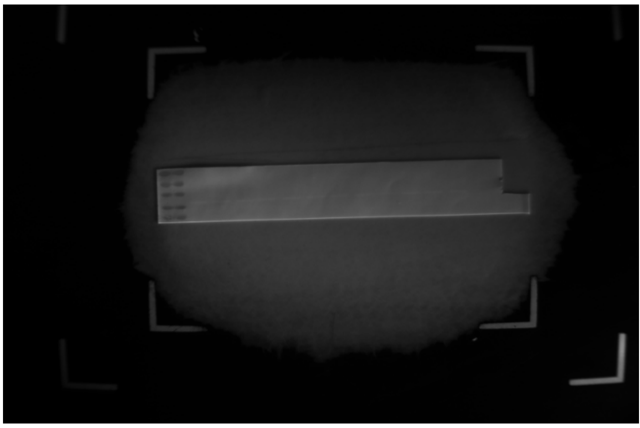

The exposure time of Figure 2A - GAPDH is 1s, so we cannot provide a membrane with clear edges.

Figure 5A: Mouse monoclonal to Tnfaip2（73 kDa）

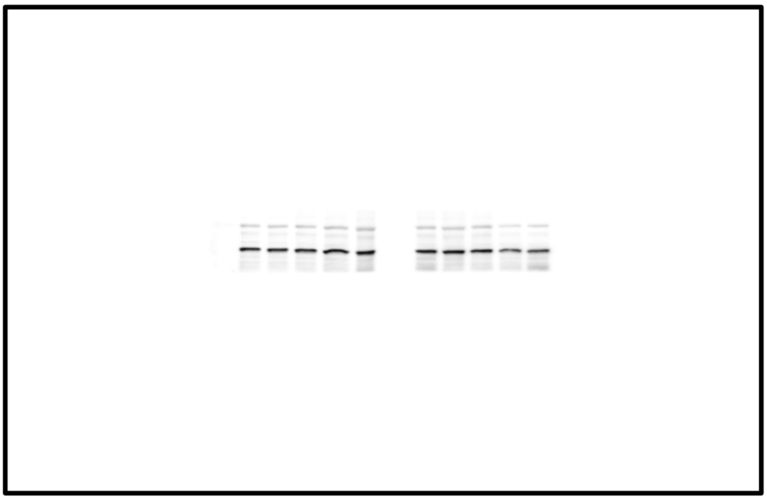

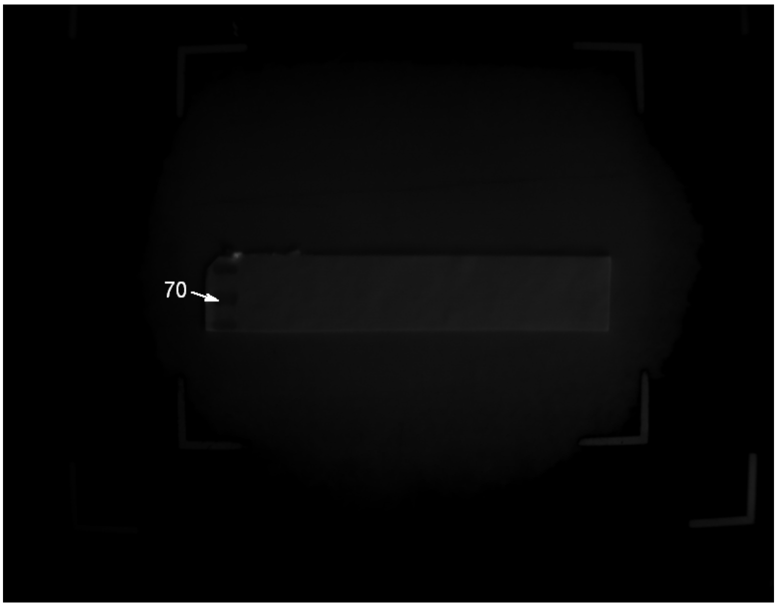

Figure 5A: Rabbit monoclonal to GAPDH（37 kDa）

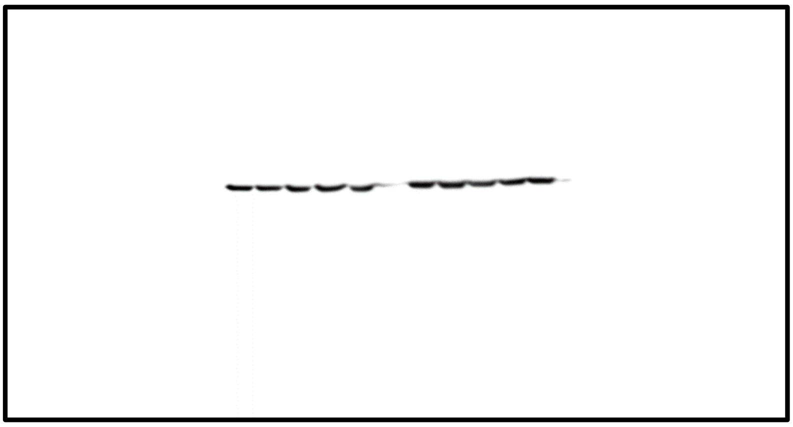

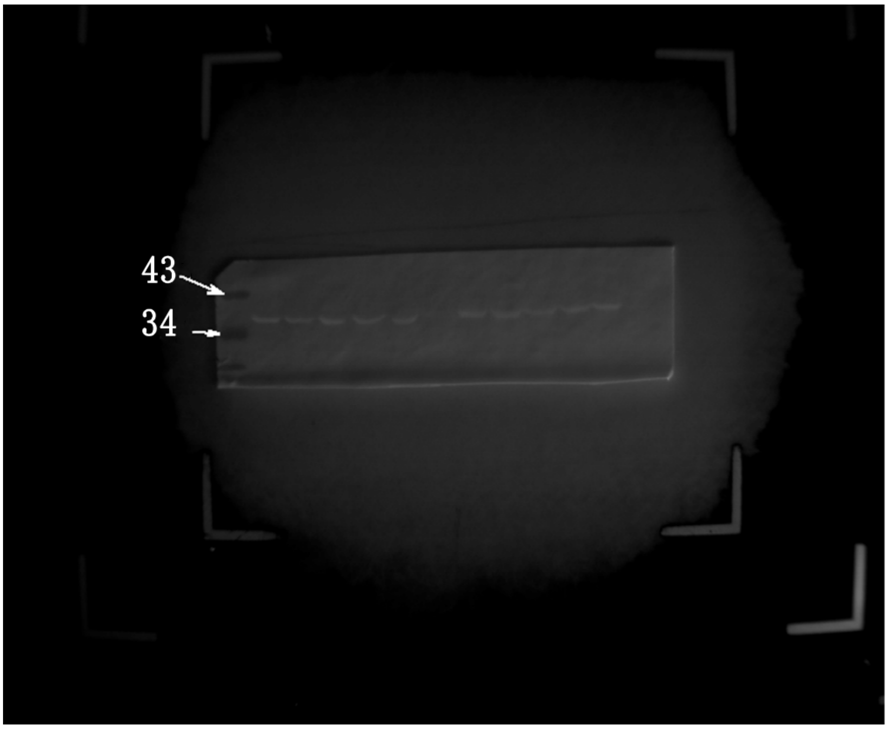

Figure 5C: Rabbit monoclonal to RPL26（20 kDa）

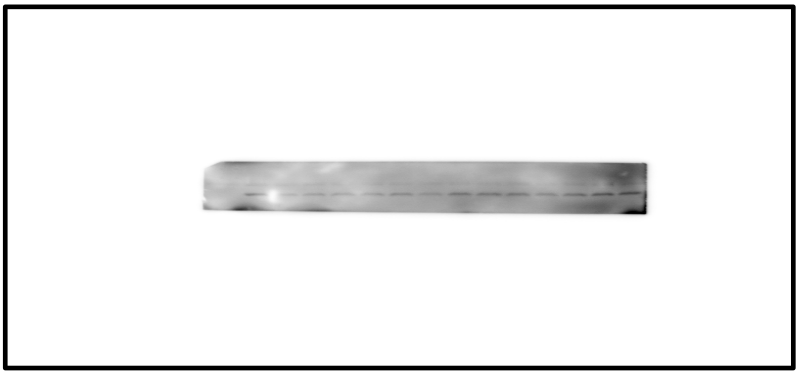

Figure 5C: Rabbit monoclonal to GAPDH（37 kDa）

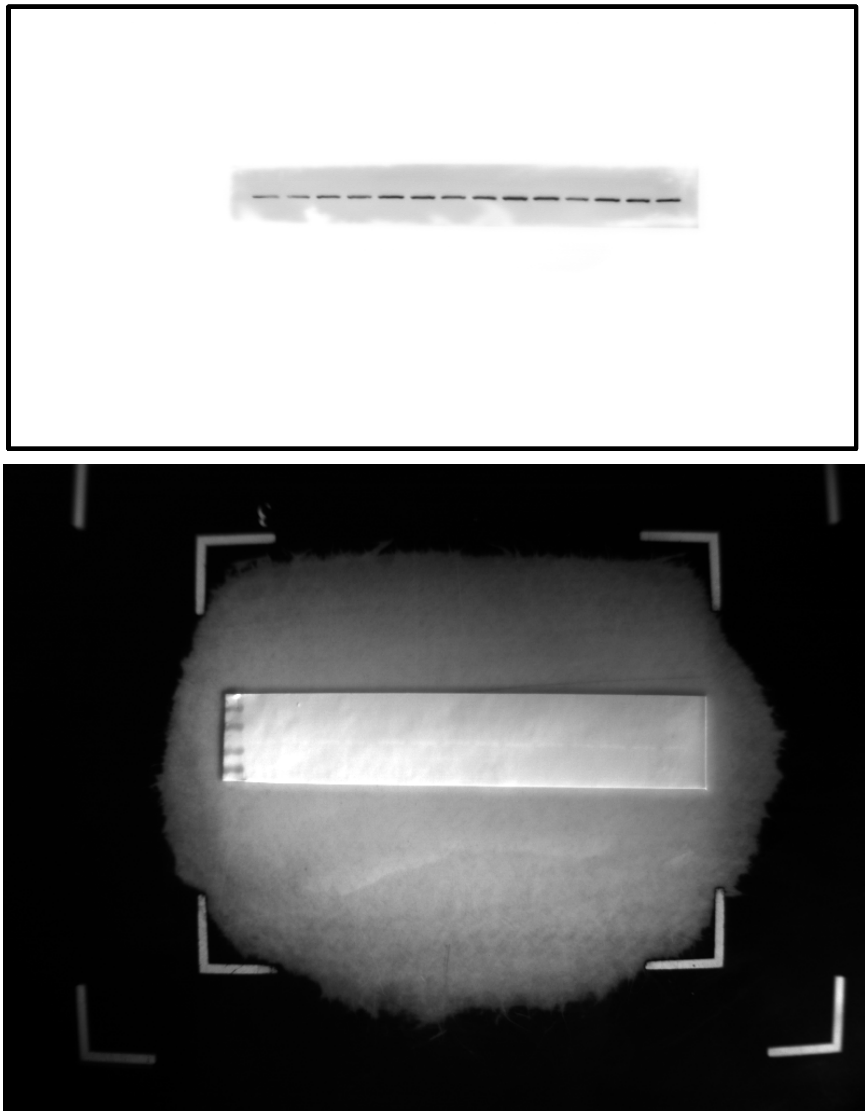

Figure 5E: Rabbit monoclonal to ufm1 （Conjugated ufm1）

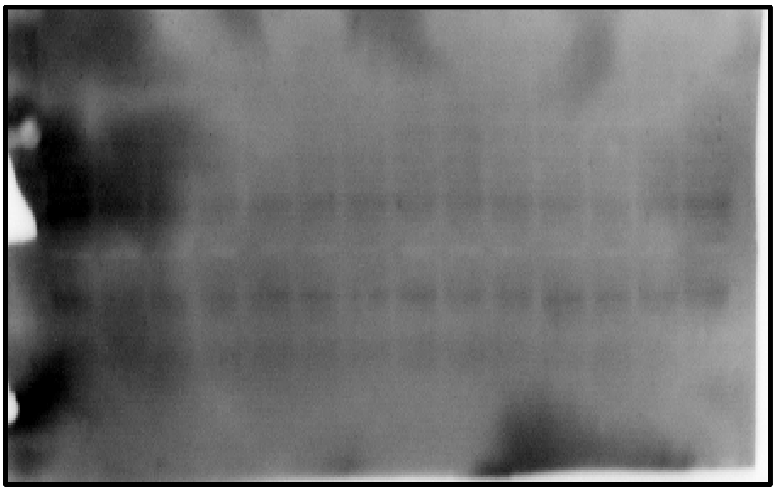

Figure 5E: Rabbit monoclonal to ufm1 （Free ufm1 9 kDa）

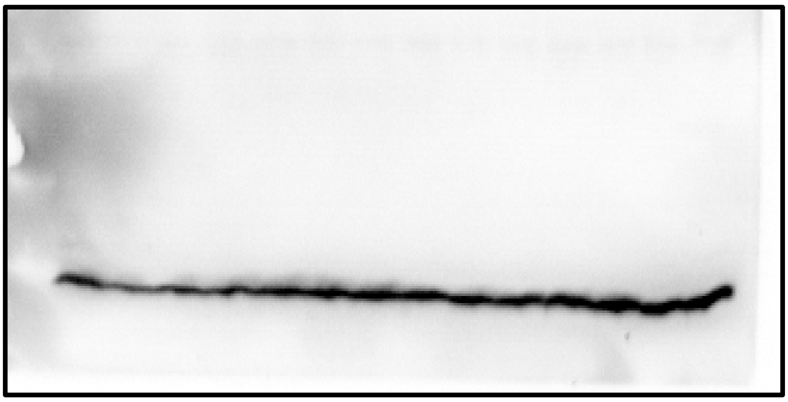

Figure 5E: Rabbit monoclonal to GAPDH（37 kDa）

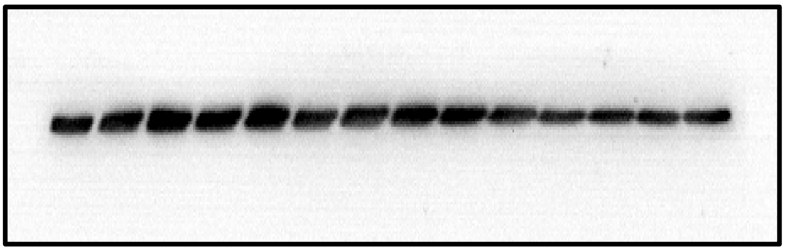

Supplement: Supplementary file 4 — Supplementary Material 4 [file 12872_2023_3563_MOESM4_ESM.pdf]
