# Supplementary material for: Screening UFMylation-associated genes in heart tissues of Ufm1-transgenic mice
Source: BMC Cardiovasc Disord. 2023 Nov 18;23:567. doi: 10.1186/s12872-023-03563-7 (PMC10657630; doi:10.1186/s12872-023-03563-7)

Figure 1 A: Rabbit monoclonal to ufm1 （Free ufm1 10 kDa）

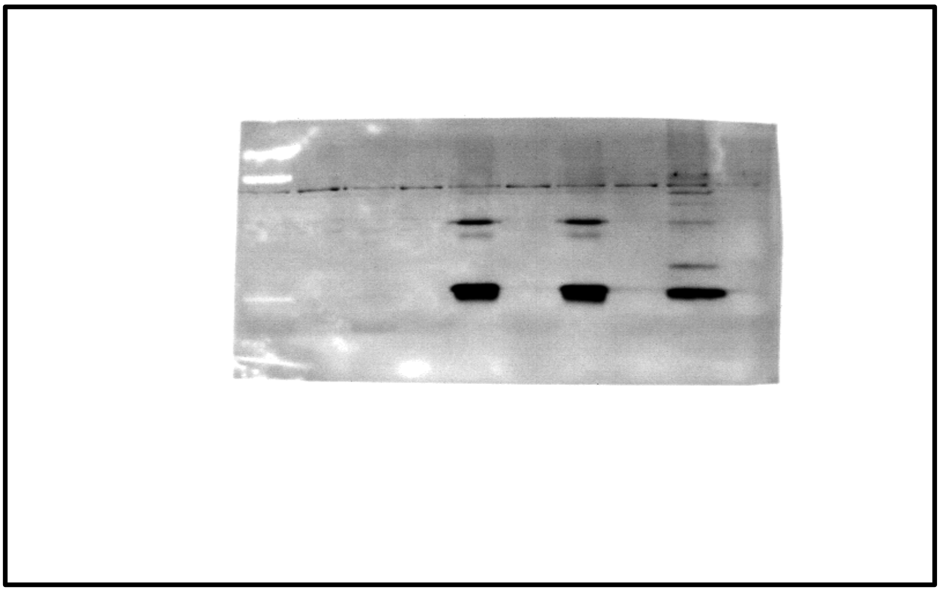

Figure 2B: Rabbit monoclonal to Ufm1 （Free Ufm1 10 kDa）

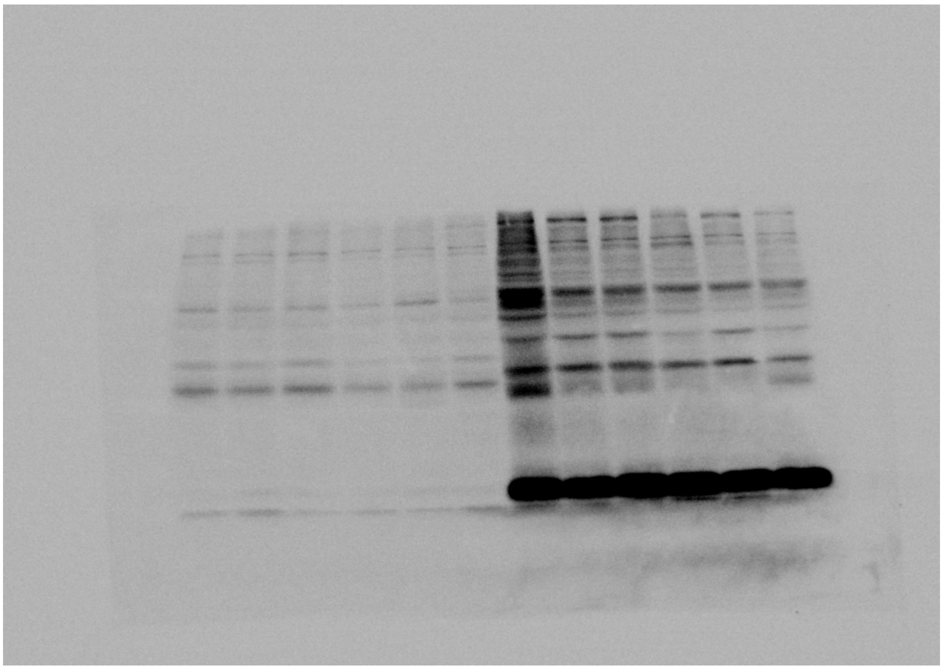

Figure 2A: Rabbit polyclonal to β -tubulin of Ufm1（50 kDa）

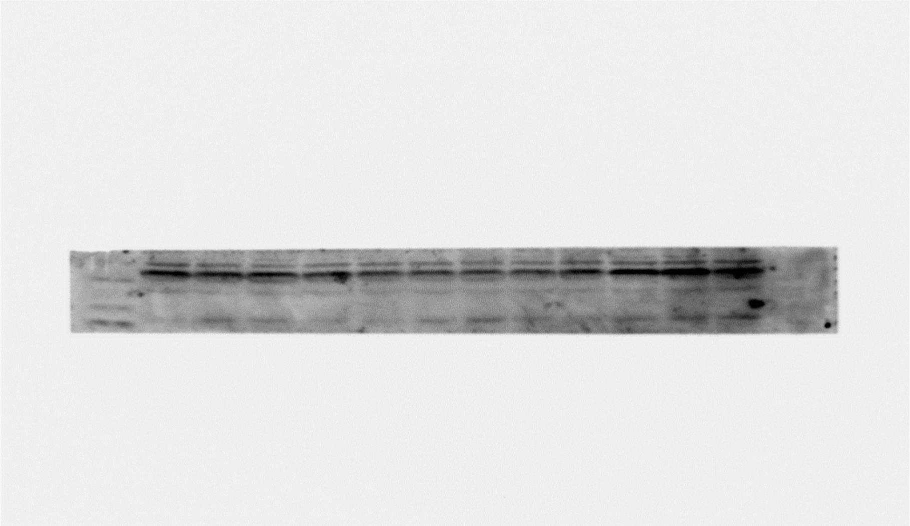

Figure 3A: Rabbit polyclonal to Uba5（45 kDa）

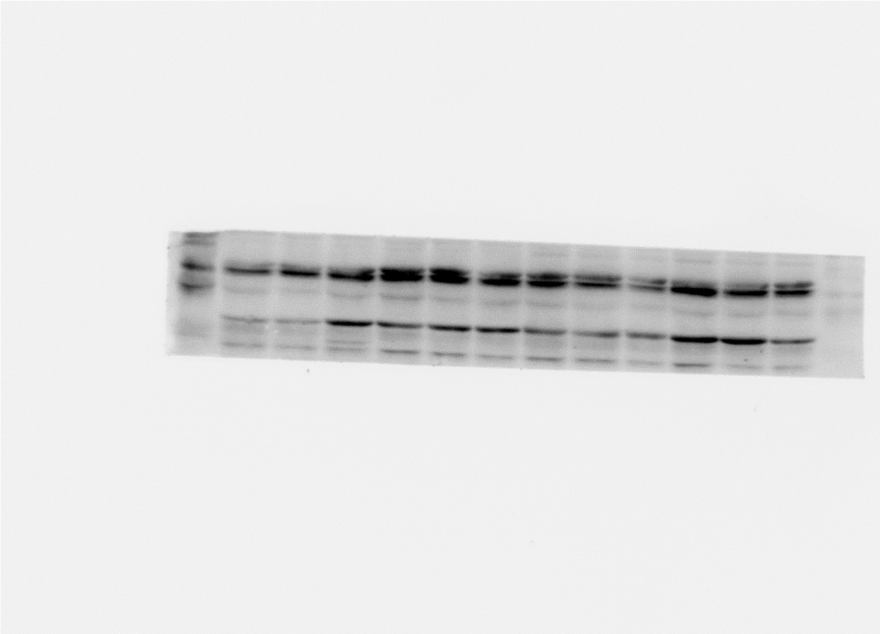

Figure 3A: Rabbit polyclonal to β-tubulin of Uba5（50 kDa）

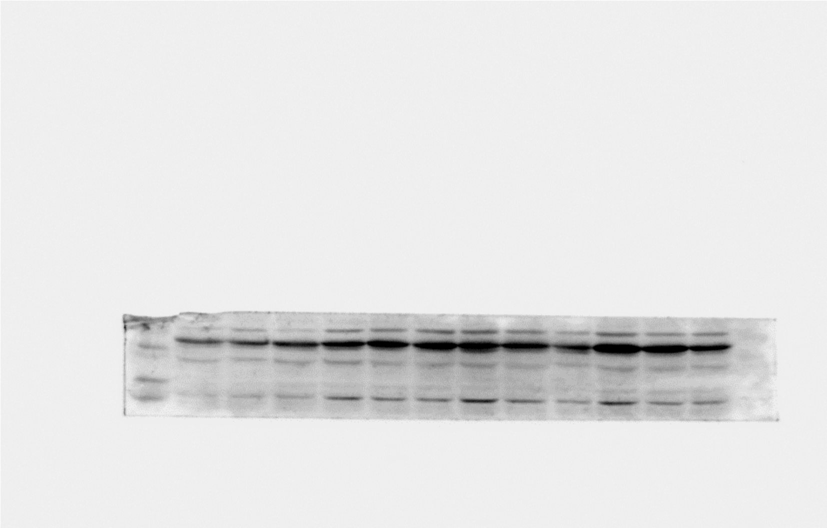

Figure 3A: Rabbit polyclonal to Ufc1（21 kDa）

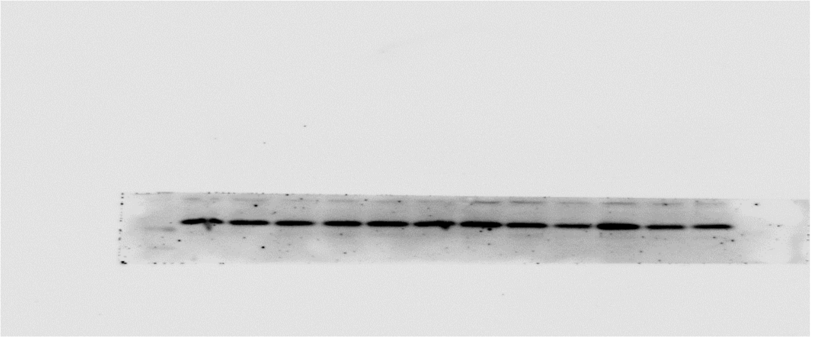

Figure 3A: Rabbit polyclonal to β-tubulin of Ufc1（50 kDa）

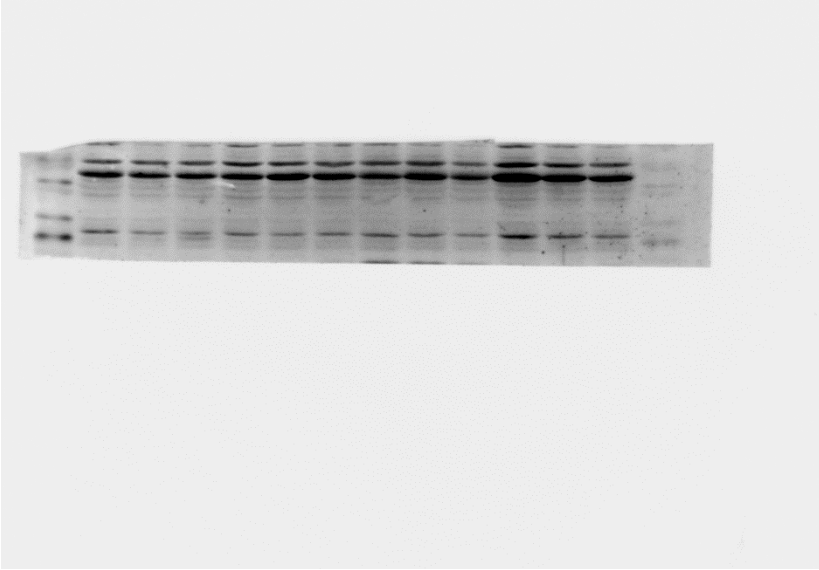

Figure 3A: Rabbit polyclonal to Ufl1（100 kDa）

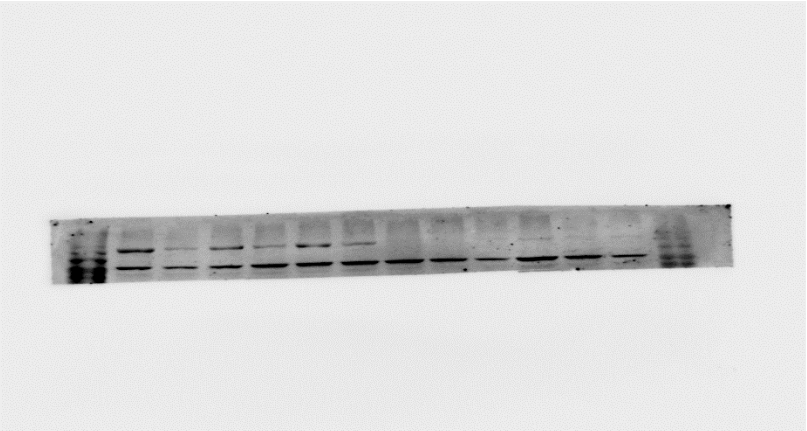

Figure 3A: Rabbit polyclonal to β-tubulin of Ufl1（50 kDa）

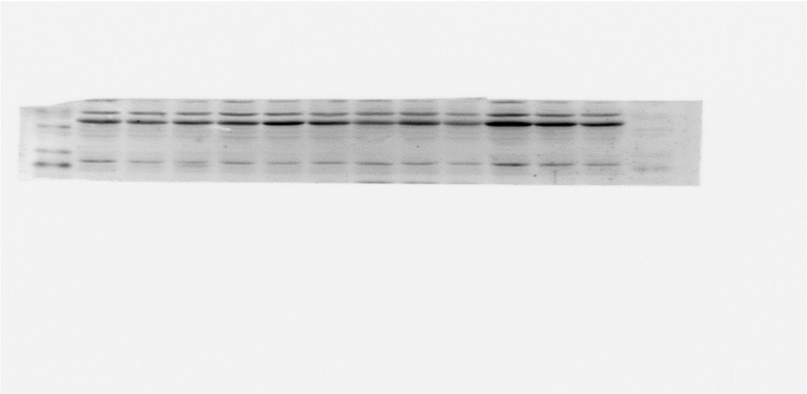

Figure 4H: Mouse monoclonal to Tnfaip2（73 kDa）

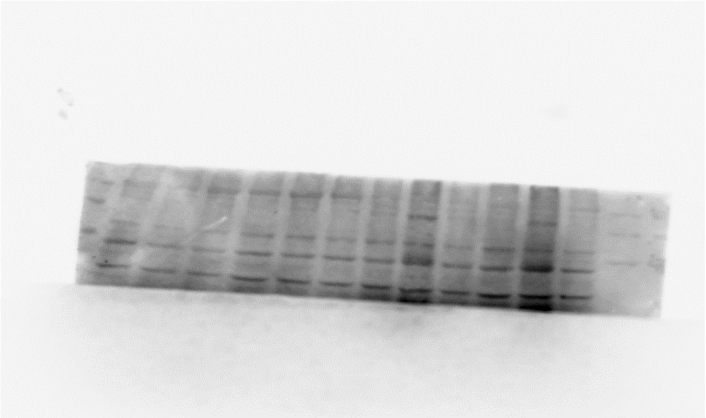

Figure 4H: Rabbit polyclonal to β-tubulin（50 kDa）

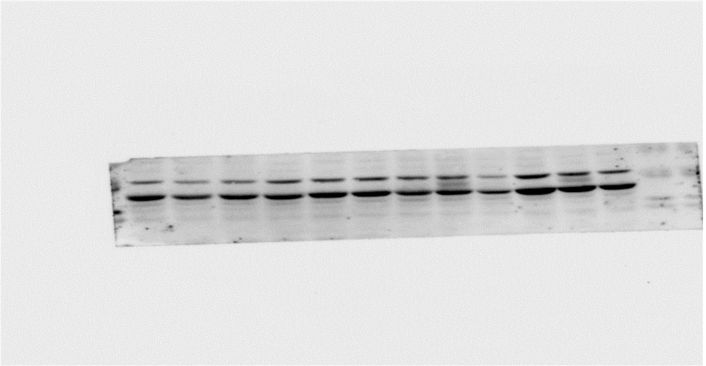

Supplement: Supplementary file 5 — Supplementary Material 5 [file 12872_2023_3563_MOESM5_ESM.pdf]
