# Supplementary figures and images for: Screening UFMylation-associated genes in heart tissues of Ufm1-transgenic mice
Source: BMC Cardiovasc Disord. 2023 Nov 18;23:567. doi: 10.1186/s12872-023-03563-7 (PMC10657630; doi:10.1186/s12872-023-03563-7)

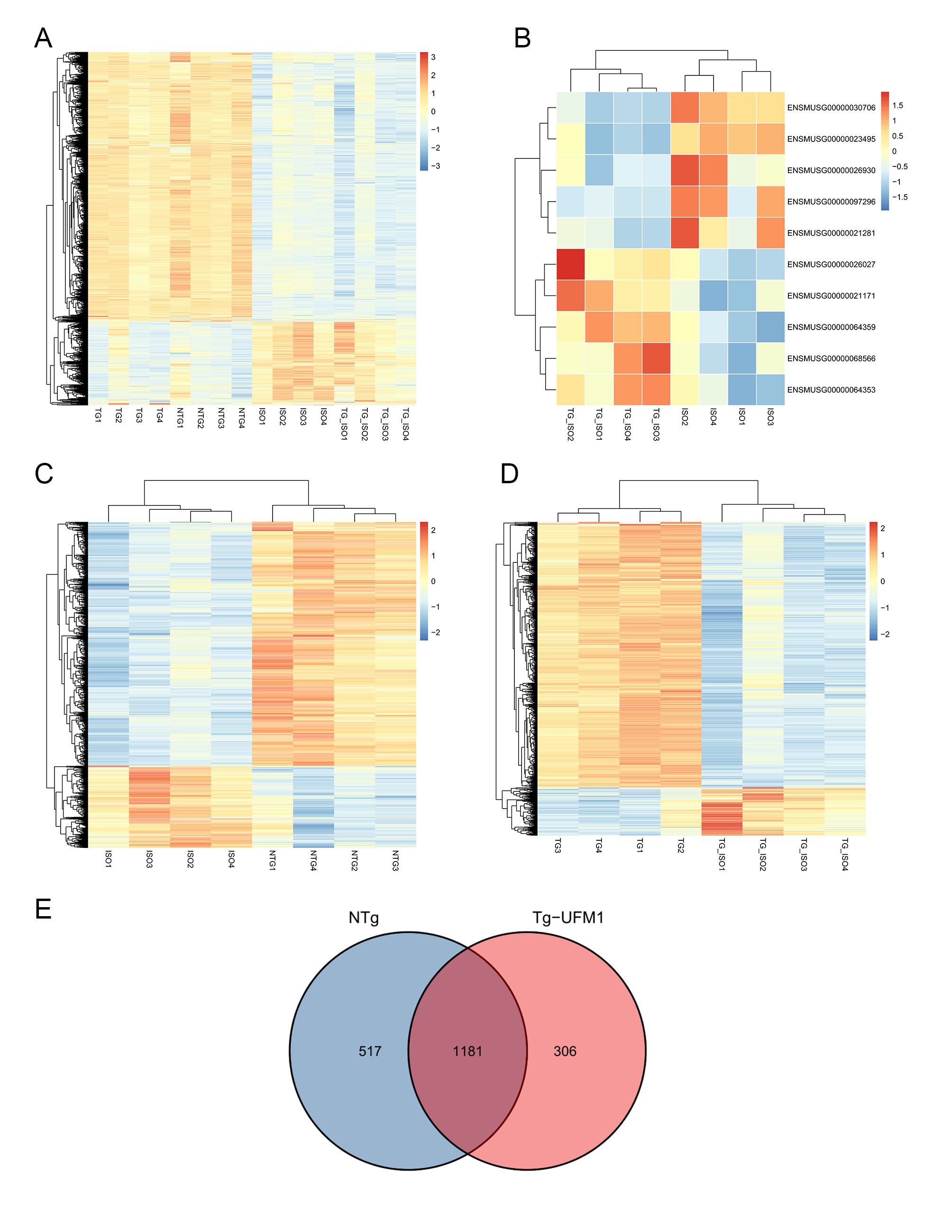

Supplement: Supplementary file 6 — Supplementary Material 6 [file 12872_2023_3563_MOESM6_ESM.tif]
