# Supplementary material for: Screening UFMylation-associated genes in heart tissues of Ufm1-transgenic mice
Source: BMC Cardiovasc Disord. 2023 Nov 18;23:567. doi: 10.1186/s12872-023-03563-7 (PMC10657630; doi:10.1186/s12872-023-03563-7)
Supplement: Supplementary file 7 — Supplementary Material 7 [file 12872_2023_3563_MOESM7_ESM.docx]

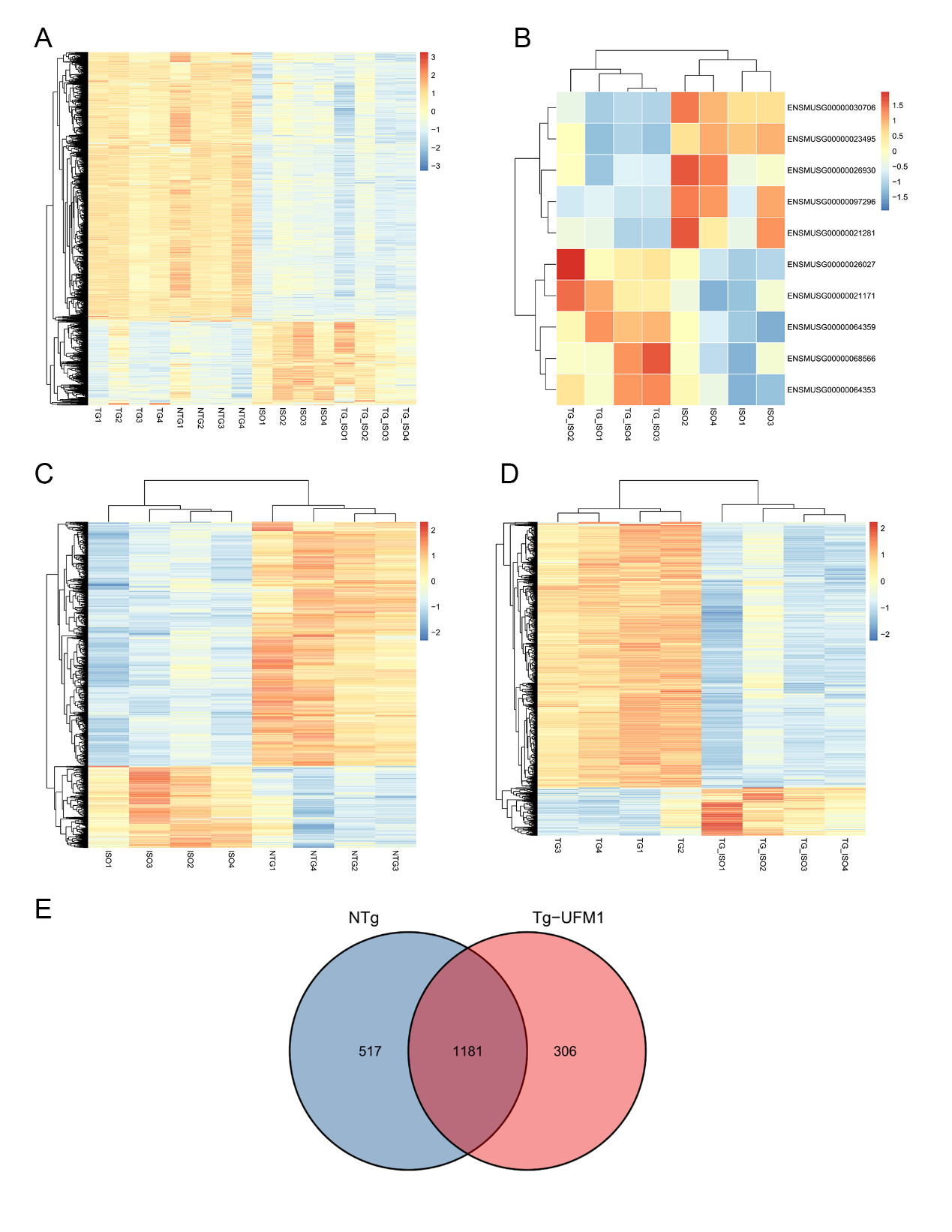


Supplemental Figure 1: Screening of UFMylation-associated genes in heart tissues using transcriptomic analysis. (A) The overall differentially expressed genes (DEGs) in heart tissues of mice as indicated. (B) DEGs between Tg and NTg mice treated with ISO. (C-E) DEGs of ISO-induced hypertrophic hearts in NTg (C) and Tg (D) mice. Overlapping analysis between DEGs described in (C) and (D), and the Venn diagram is shown in (E).
